# Reliable and Accurate Release of Micro-Sized Objects with a Gripper that Uses the Capillary-Force Method

**DOI:** 10.3390/mi8060182

**Published:** 2017-06-08

**Authors:** Suzana Uran, Riko Šafarič, Božidar Bratina

**Affiliations:** Laboratory of Cognitive Systems in Mechatronics, Faculty of Electrical Engineering and Computer Science, University of Maribor, SI-2000 Maribor, Slovenia; suzana.uran@um.si (S.U.); riko.safaric@um.si (R.Š.)

**Keywords:** micro-objects, one-finger gripper, gripping, releasing, capillary force

## Abstract

There have been recent developments in grippers that are based on capillary force and condensed water droplets. These are used for manipulating micro-sized objects. Recently, one-finger grippers have been produced that are able to reliably grip using the capillary force. To release objects, either the van der Waals, gravitational or inertial-forces method is used. This article presents methods for reliably gripping and releasing micro-objects using the capillary force. The moisture from the surrounding air is condensed into a thin layer of water on the contact surfaces of the objects. From the thin layer of water, a water meniscus between the micro-sized object, the gripper and the releasing surface is created. Consequently, the water meniscus between the object and the releasing surface produces a high enough capillary force to release the micro-sized object from the tip of the one-finger gripper. In this case, either polystyrene, glass beads with diameters between 5–60 µm, or irregularly shaped dust particles of similar sizes were used. 3D structures made up of micro-sized objects could be constructed using this method. This method is reliable for releasing during assembly and also for gripping, when the objects are removed from the top of the 3D structure—the so-called “disassembling gripping” process. The accuracy of the release was lower than 0.5 µm.

## 1. Introduction

During micro-assembly, individual micro-sized objects are picked up and placed at a desired destination using a gripping tool. In contrast to macro-assembly, the challenge of assembling micro-sized objects not only entails gripping the objects, but also releasing the objects at a desired location. The main difficulty is at the micro-size level, when surface forces (Van der Waals force, electrostatic force, capillary force …) start to dominate the volumetric gravitational force. Because of this, releasing micro-sized objects using the force of gravity cannot be reliably used. Macro-assembly approaches, in general, cannot simply be downsized and used for micro-assembly. Accordingly, intensive research in the area of micro-assembly using micro-grippers has been done in the last decade. Many researchers from different fields have contributed research on micro-grippers. This has led to a great variety of approaches and terminology.

Before continuing with our overview, the following facts should be noted. In spite of the differences between macro and micro-assembly, the terminology from macro-assembly is still frequently used for micro-assembly in the research community. For instance, micro-object manipulation is performed by micro- or nano-precision robots (robot arms) using a tool called a micro-gripper or a (micro) gripping tool, even though neither the micro-robots nor the micro-grippers resemble robot arms or grippers. Another frequently encountered term for a micro-gripper, originating from laboratory practice, is a variant of the word tweezers, for instance: optical tweezers. Also, in this case, the word tweezers describes only the principle of gripping objects. Next, micro-assembly, according to Lambert [1], deals with objects in the range from 1 μm to 1 mm in size. This is such a wide range of object sizes that a single micro-assembly approach would not be able to deal with such a large range. This is definitely true for the approach presented here. Therefore, in the following article, the range of object sizes from 1 μm to 1 mm is split into two regions, as follows. The first region covers spherical objects with diameters in the range of 1 μm to 100 μm and is called the region of micro-sized objects. The second region covers objects with diameters in the range of 100 to 1000 μm (1 mm) and is called the region of sub-millimetre-sized objects. Our overview of micro-gripper approaches focuses on the approaches used for micro-sized objects. But some of the important micro-gripper approaches for sub-millimetre-sized objects are also included in the overview, for the sake of greater completeness. In the overview, the micro-gripper approaches are first classified by the size of the micro-objects. Afterwards, they are further classified by the type of micro-gripper, and the force used in manipulating objects. The micro-grippers are classified into three categories according to their appearance: micro-grippers with two or more fingers (2 + F), one-finger micro-grippers (1F), and traps (T). Micro-grippers with two or more fingers are basically downsized macro-object grippers. Friction or mechanical coupling may be used to grip the object, and gravity is used to release the object. When an object is gripped, it is held between both fingers, which also puts force on the object. At least two fingers are needed to ensure the stability of the grip.

One-finger grippers modify the resultant force acting on the sub-millimetre or micro-sized object during gripping and releasing procedures. In order to grip, the sum of attractive forces (gripping force) between the finger and the object must be greater than the sum of pulling/pushing forces between the object and the releasing surface (releasing force). The gripping force between the finger and the object must be lower than the releasing force during the releasing procedure. The attractive forces could be van der Waals forces between the object and the finger, capillary forces, pneumatic forces (sucking), mechanical coupling forces, and so on. The pulling/pushing forces could be gravity forces, van der Waals or capillary forces between the object and the releasing surface, repulsive forces due to hydrophobicity, pneumatic forces (blowing), inertial forces (vibration), and so on. There may be several forces involved in the gripping or releasing, but usually only one of the forces listed in the previous two sentences is dominant. The releasing of the object may be achieved either by reduction of the gripping force between the finger and the object while the releasing force remains constant, or by keeping the gripping force constant while increasing the releasing force.

The last category of grippers is called traps. In this case, the micro-sized object is trapped because it is enclosed by the gripper (freeze tweezers, optical tweezers). Consider, for example, optical tweezers, where the gripping force on the micro-object is a result of laser beams enclosing the object. Surface forces between the micro-gripper and the micro-sized object do not exist. Therefore, no releasing force is needed. Optical traps are used for manipulating nano-sized (1 nm to 100 nm), sub-micrometre-sized (100 nm to 1 μm) and micro-sized objects up to 10 μm, or even more. A selection of micro-assembly approaches are shown in Table 1.

The overview of micro-assembly approaches in Table 1 shows both active and passive approaches, as well as a wide variety and combination of gripping/releasing forces that are used with the approaches presented in this article. Active approaches use external energy to produce the pneumatic forces, laser beam forces, and capillary forces due to dropwise or thin film condensation, inertial forces, etc. Passive approaches use gravity forces, van der Waals forces, etc. It can be seen that for larger sub-millimetre-sized objects, gravity can be used as a releasing force when combined with actions that intentionally reduce the surface force. With the development of approaches and increased knowledge and experience, the van der Waals force can also be successfully used as a releasing force for sub micro-sized objects. The capillary force has been observed to be a very strong surface force with the ability to grip sub-millimetre and micro-sized objects.

The capillary force is influenced by intrinsic and boundary parameters [2]. The intrinsic parameters are related to the physical properties of the liquid that produce the capillary force (the density of the liquid and its surface energy). The boundary parameters are related to environmental characteristics, surface characteristics and electrochemical interactions. The environmental characteristics are determined by the working environment (temperature, relative humidity and pressure). The surface characteristics are determined by the geometry (curvature radius, dimensions and roughness) of the objects involved that are affected by the capillary force. Electrochemical interactions define the interaction parameters (electrostatic charge distribution, contact angle, line tension) between the objects and the liquid. The work area setup for the capillary gripping/releasing method is controlled by environmental (temperature) and surface characteristics (curvature radius and dimensions) to produce a variable capillary force.

The latest developments in capillary force approaches show that the capillary force can be dynamically controlled (dropwise condensation) by temperature, and can be used for gripping [14]. On the basis of controlled temperature, it is believed that the liquid volume can be controlled sufficiently when using tiny amounts of liquid, so that the use of the capillary force can be extended to manipulating micro-sized objects. Moreover, the approach presented in the article shows that by temperature control, a capillary bridge can be built or eliminated by condensation/evaporation wherever it is suitable, resulting in the successful gripping and releasing of micro-objects.

The remainder of the article details the development of a one-finger gripper based on the capillary force, produced from a thin layer of condensed water. This gripper is able to reliably grip and release micro-sized objects (spheres with diameters of 5–60 µm) using the capillary force for both actions: gripping and releasing. We do not use the dropwise method on the tip of the one-finger gripper, but a thin layer of water from which the water meniscus between the object and the gripper (gripping procedure) or between the object and the releasing surface (releasing procedure on the base surface), or between two or more objects (releasing procedure during 3D construction) is produced. Moreover, the proposed method also allows “disassembling gripping”, which is like the process of gripping micro-sized objects that are on top of each other, but with a smaller object under the larger object. This technique is particularly important during the practical execution of building 3D structures, when the micro-sized object has to be removed from one part of the 3D structure and placed onto another part of the same 3D structure. The releasing accuracy of the released micro-object for the presented technique is lower than 0.5 µm.

The reason why the proposed method is crucial is that it is useful for building 3D constructions, due to its reliable releasing and reliable “disassembling gripping”. Not all of the reviewed techniques presented in this section can be used for 3D assembling, with the exception of techniques [13,14,15] and possibly [11,12]. However, without exception, none of the reviewed techniques in this section can be used for 3D disassembling. Possible applications for this method are assembling and disassembling techniques, where micro-sized or sub-millimetre-sized 3D structures (like gear-boxes, bearings, micro-machines, micro-electromechanical systems etc.) are built out of micro-sized objects.

## 2. Materials and Methods

### 2.1. Materials

SiO_2_ (amorphous) spheres with diameters of 2–10 μm, 10–30 μm, and 50–100 μm were obtained from PolySciences, Inc. (Warminster, PA, USA). Polystyrene spheres (diameter 30–40 µm) were purchased from Kisker Biotech GMBH & Co. KG (Steinfurt, Germany). The tips of the one-finger gripper were made of golden wire (Premion 99.995%, as a metal basis), with a diameter of 50 μm, and were purchased from Alfa Aeser GmbH & Co. KG (Karlsruhe, Germany).

### 2.2. Laboratory Set-Up

**Figure 1:**
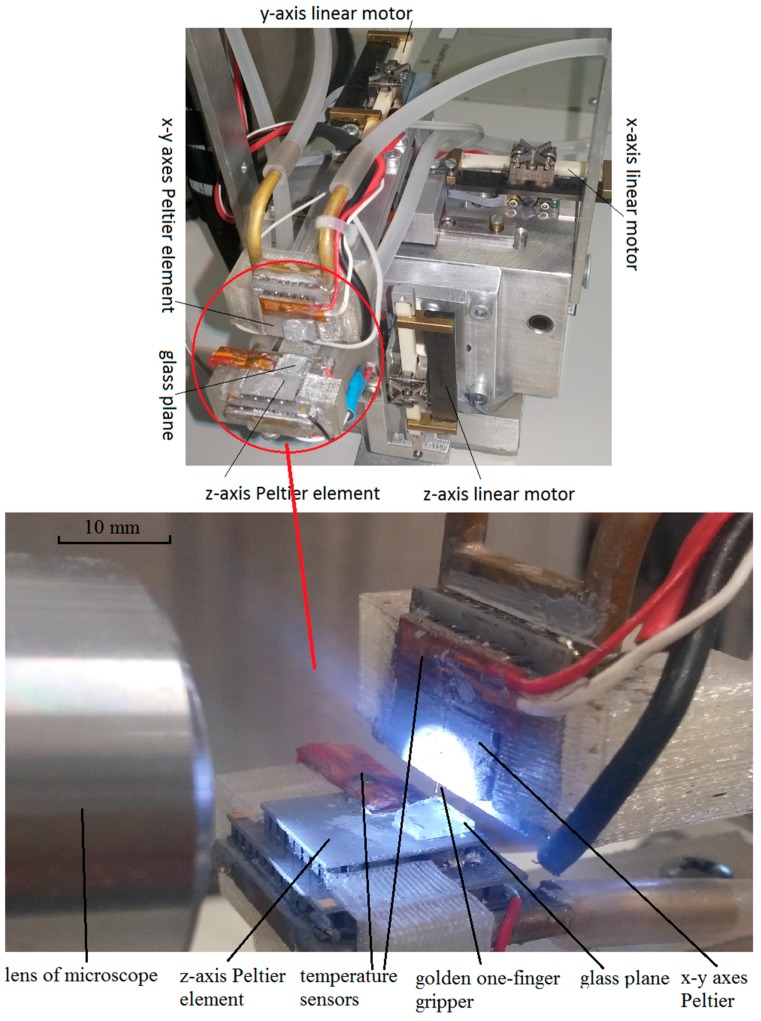
Nano-precision robotic system.

A nano-precision robotic system [13,20] was used to move the gripper (Figure 1). The robotic workspace has a volume of approximately 1.5 cm^3^. The tip position of each axis in a closed-loop position had an accuracy of ±61 nm with a magnetic incremental encoder, or 3.9 nm (the smallest step on the piezo-electric motor) in an open-loop without the encoder.

**Figure 2:**
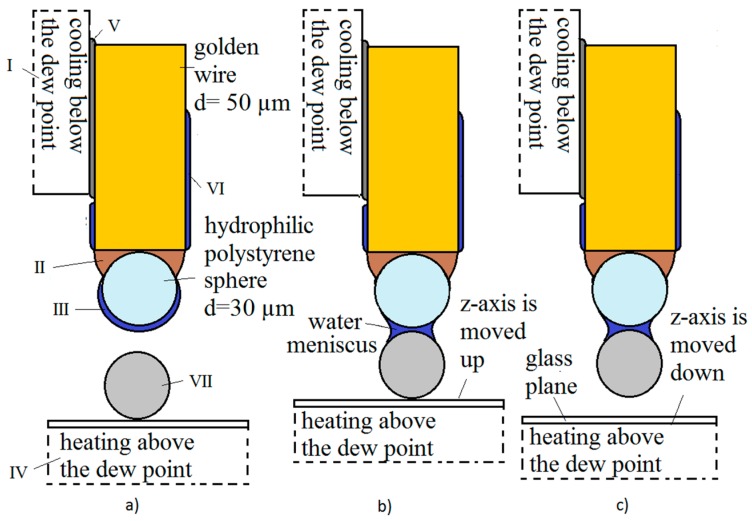
Gripping procedure using the capillary force method based on a thin layer of condensed water: (**a**) a thin layer of water was created on the tip; (**b**) a water meniscus was created between the tip and the object; (**c**) the object gripped to the tip. (I—Peltier element *x*-axis; II—hydrophobic epoxy adhesive; III—condensed layer of water; IV—Peltier element *z*-axis; V—heat conducting paste; VI—thin layer of condensed water; VII—spherical micro object, *d* = 5–60 µm).

The gripping/releasing hardware was made up of the following (see Figure 2a): an *x*-*y* Peltier element on the tip of the *x*-*y* axes of the robotic mechanism, gold wire (diameter of 50 µm) glued to the *x*-*y* Peltier with heat conducting paste (silver based), a polystyrene sphere (diameter of 30 µm) glued to the golden wire with a hydrophobic epoxy adhesive, and a *z*-axis Peltier element covered with a plate of amorphous glass (SiO_2_). The hydrophobic epoxy prevented the condensed water on the golden wire from moving to the polystyrene sphere. Each Peltier element had a temperature sensor; therefore it could control the surface temperature from −45 to +45 °C with a proportional–integral–derivative (PID) controller.

A more precise description of the nano-precision robotic system can be found in [13,20]. These include the position control system, the mechanical design, man-machine communications, and other technical details.

### 2.3. Gripping/Releasing Method

The gripping/releasing methods used a thin film of condensed water from atmospheric air moisture with a pressure of 1 bar. This film was situated between the micro-objects and the tip of the one-finger gripper or glass plane surface. This produced a capillary force between them. A thin film was created in a few seconds (approximately 1–2 s for micro objects sized between 10–50 µm), after the temperature of the object was decreased to just below the dew point temperature. A golden rod was used to make a thin film of water in as short a time possible. Gold was used because it has good thermal conductivity [318 W/(m·K)] and does not corrode. The dew point temperature was approximately between 10–20 °C with normal atmospheric relative humidity between 30–60%.

**Figure 4:**
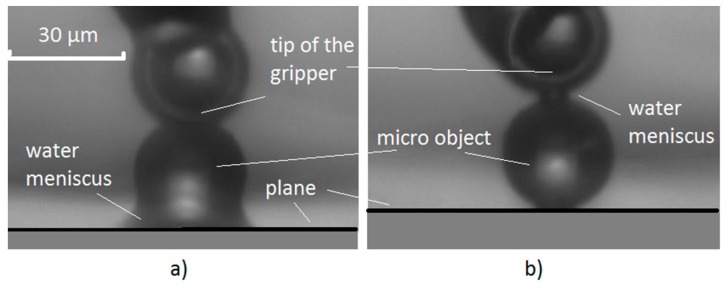
(**a**) Water meniscus is formed between the glass plane and a polystyrene micro-object with a radius of 30 µm; (**b**) water meniscus is formed between the tip of the gripper and a polystyrene micro-object.

**Figure 8:**
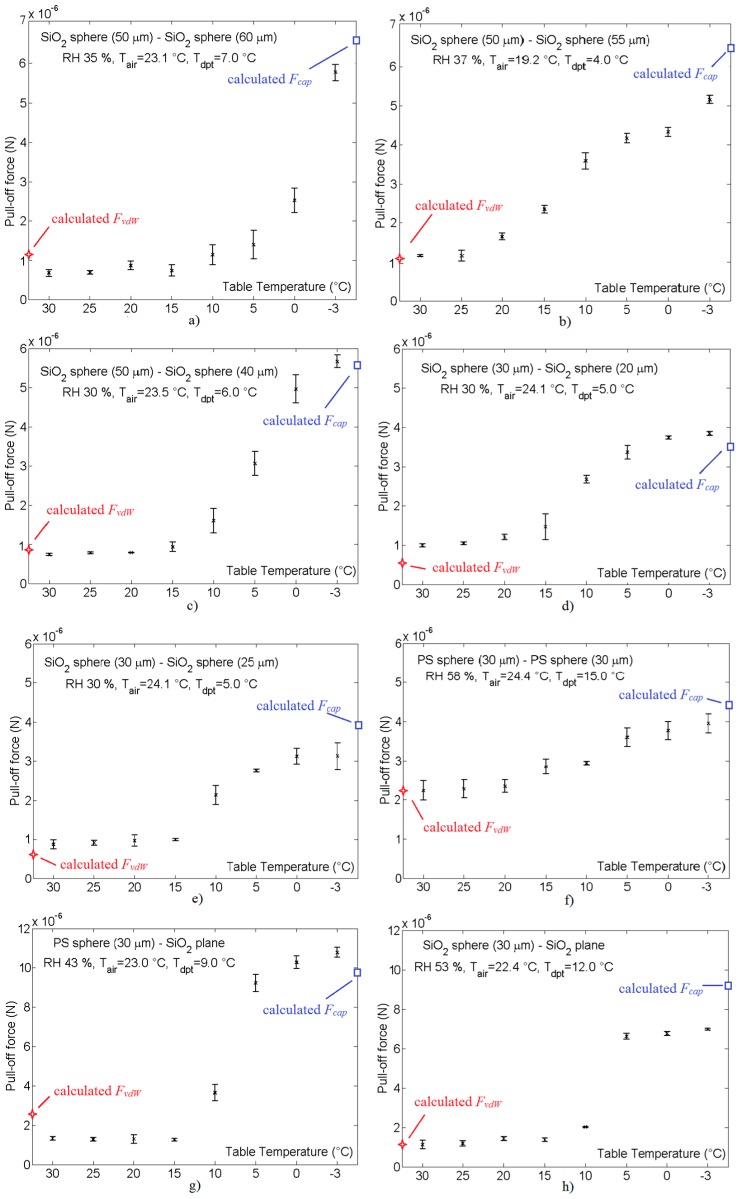
Measurements of the pull-off forces with their standard deviations vs. the table temperature between spherical micro-sized objects with different diameters, materials and humidity levels (RH): (**a**) SiO_2_ sphere (*d* = 50 μm)-SiO_2_ sphere (*d* = 60 μm); (**b**) SiO_2_ sphere (*d* = 50 μm)-SiO_2_ sphere (*d* = 55 μm); (**c**) SiO_2_ sphere (*d* = 50 μm)-SiO_2_ sphere (*d* = 40 μm); (**d**) SiO_2_ sphere (*d* = 30 μm)-SiO_2_ sphere (*d* = 20 μm); (**e**) SiO_2_ sphere (*d* = 30 μm)-SiO_2_ sphere (*d* = 25 μm); (**f**) Polystyrene sphere (*d* = 30 μm)-Polystyrene sphere (*d* = 30 μm); (**g**) Polystyrene sphere (*d* = 30 μm)-SiO_2_ plane; (**h**) SiO_2_ sphere (*d* = 30 μm)-SiO_2_ plane. (Remark: The sizes of the spheres are their diameters. The temperature is measured approximately 6 mm away from the place where the micro-sized object and the tip of the gripper touch each other, so the actual temperature of the object and the tip of the gripper may differ by 2–3 °C).

The gripping procedure is shown in Figure 2:

- The temperature of the *x*-*y* Peltier element was lowered below the dew point. This created a thin layer of condensed water on the gripper’s tip (polystyrene sphere) in 2–3 s (Figure 2a).
- The tip, with the thin layer of water, was moved so close to the micro-object that it slightly touched it. Immediately, a water meniscus was created between the tip and the object (Figure 2b and Figure 4b). This created a capillary force between the object and the tip. The object was then warmed through the glass plane, to a temperature that was a bit above the dew-point by the *z*-axis Peltier element (the water accumulated in the meniscus completely evaporated), so that only the van der Waals force existed between the object and the glass plane (plate).
- The capillary force between the object and the tip was greater than the van der Waals force between the glass plane and the object (see Figure 8). Therefore, the object gripped to the tip when the *z*-axis was moved down (Figure 2c).

**Figure 3:**
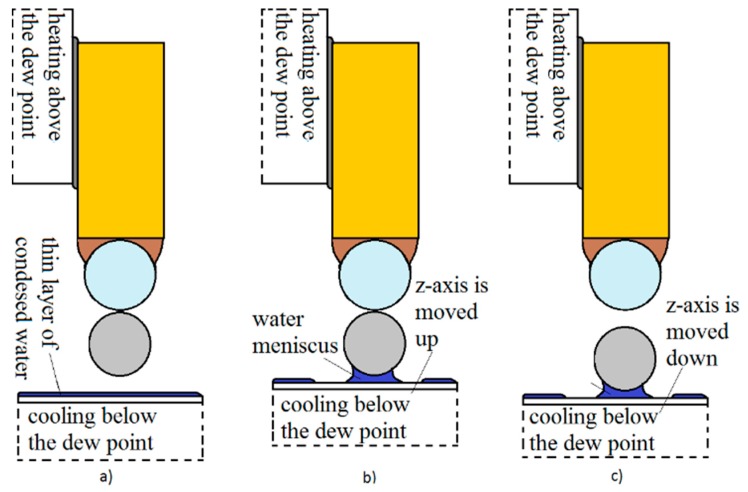
Releasing procedure using the capillary force method based on a thin layer of condensed water: (**a**) the water from meniscus evaporated; (**b**) a water meniscus between the object and the glass plane was created; (**c**) the object was released from the tip.

The releasing procedure is shown in Figure 3:

- The temperature of the *x*-*y* Peltier element was increased so that the temperature of the tip of the gripper was above the dew point. The water from the meniscus evaporated in 2–3 s (Figure 3a). Consequently, only the van der Waals force remained between the gripper’s tip and the micro-object.
- The temperature of the *z*-axis Peltier element was decreased at the same time. The glass plane reached slightly below the dew point temperature in 2–3 s and a thin layer of condensed water from the surrounding air was created, at a pressure of 1 bar. The *z*-axis, along with the glass plane and the thin layer of water, was moved up toward the gripped micro-object, so that it slightly touched it. Immediately, a water meniscus between the object and the glass plane was created (Figure 3b and Figure 4a).
- The *z*-axis was moved down and the object was released from the tip (Figure 3c). This was caused by the capillary force between the glass plane and the object, the force being greater than the van der Waals force between the gripper’s tip and the object.

The complete griping/releasing procedure described by Figure 3 and Figure 4 is shown in Video S1.

Before moving an object, a test was conducted to assure that the sphere was rigidly fixed to the gold wire. This was done by carefully touching the glass plane with the sphere, which was glued to a gold wire. Then the tip of the gripper was pushed slightly to the left and right on the glass plane to determine if there was any movement of the glass sphere in the glue. Calibration of the tip was achieved by positioning the tip to one of the corners of the glass plane; this marked the starting position (*x*, *y* and *z*).

The gripping and releasing of micro-sized objects is done using a glass or polystyrene sphere on the tip. To do this, it is necessary to calibrate the dew point. This is necessary because the relative humidity in the air, the surrounding temperature and the type of tip (material) can be different every time. Thus, before trying to move an object for the first time, a simple test is conducted with the prepared tip. Temperatures measured on the Peltier elements are important. This is where water from the air condenses onto the surfaces of the tip and the plane. The difference between these two temperatures determines the gripping or releasing time and procedure. An additional temperature offset is assumed because the tip (gold wire) is mounted slightly over the Peltier’s edge, where the temperature is conducted through the paste, on through the gold wire, to the end of the tip (sphere). This offset makes it so that the temperature of the tip’s end and the Peltier surface are usually ever the same, due to surrounding disturbances (heat losses). On the other hand, the glass plane surface is directly mounted onto the Peltier. Thus it has smaller temperature losses and is similar to the Peltier’s temperature. The following was done to determine the best temperature for gripping: first we set the temperatures on both sides to 30 °C and waited for both surfaces to become completely dry. Then the tip’s temperature was incrementally lowered by steps of 5 °C, at each increment we tried to grip the object. An additional ±1 °C correction in temperature was made around the reliable point of gripping. Secondly, we determined the temperature point for releasing the object. We dried the tip and started to cool it down the surface plane by increments of 5 °C. Similarly, additional corrections (±1 °C) were made around the reliable point of releasing. Usually both temperatures were complementary, meaning that we additionally set the tip’s temperature to between 5 °C and 20 °C on the plane surface for gripping, and vice versa for releasing. With properly determined temperatures, under local environmental conditions, a reliable gripping and releasing could be achieved, which could be upgraded from manual to automated mode (assembly line).

### 2.4. Pull-Off Force Measurement Method

**Figure 5:**
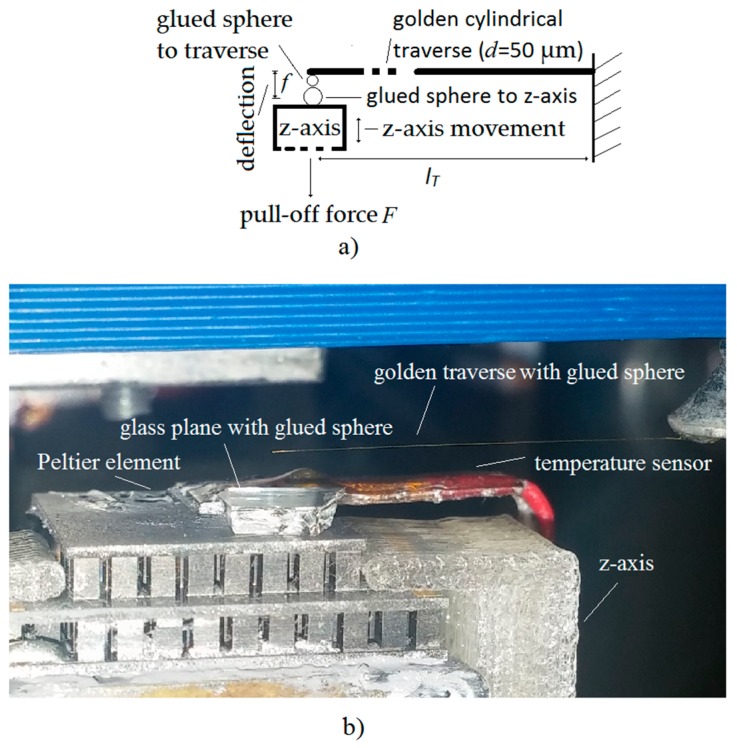
Measurements of the pull-off force: (**a**) description of a pull-off method; (**b**) lab test-bed for pull-off method.

Figure 5 shows the measurement method for the pull-off force. The pull-off force *F* attracts micro-sized objects when they have been put into contact. If the lower object is pulled down by the force *F* with the *z*-axis (see Figure 5) then the traverse starts to deflect with the distance *f*. The objects are “attached” together during the deflection, due to the attraction force. The objects keep this position until the opposite directed traverse elastic force becomes equal to the attraction force. At that moment, the traverse tears away from the lower object towards a position of equilibrium and the deflection *f* is read. The elastic traverse is rigidly mounted onto one side of the tip of the *x*-*y* axes of the nano-precision robot system (see Figure 1), while the other side is left free. The *z*-axis is cooled down with the Peltier element to produce a thin layer of condensed water on the sphere glued to *z*-axis. The equation for calculating the pull-off force *F* of the traverse with a circular cross-section is given by Equation (1):

(1) $$F=\frac{3fE\pi d^{4}}{l_{T}^{3}64}$$

where *l_T_* is the length of the traverse, *f* is the deflection, *E* is Young’s modulus of the gold, and *d* is the diameter of the golden traverse. A more precise description of the pull-off measurement method is presented in [1,21]. The accuracy of the method is approximately ±25 nN, if the measurements are done in a vibration-free environment, and *l_T_* = 40 mm, *d =* 50 µm, *E* = 79 GPa and *f* has an accuracy of ±61 nm.

The measurement of the capillary force between two spheres is shown in Video S2.

## 3. Results

### 3.1. Lab Experiments for Releasing/Gripping a Sphere and Placing It on Top of Another Sphere

The above described gripping/releasing procedure is suitable for constructing 2D structures on a glass plate—*x*-*y* plane. To build 3D structures, it is necessary to build in a vertical dimension. Therefore, we will describe such a method, and present the lab experiments for releasing/gripping a sphere on top of another sphere:

- Releasing: Two meniscuses have to be created: one between the glass plane and the bottom sphere, and one between both spheres when releasing a micro-object on top of another one. This is done by cooling the glass plane over a longer period of time (approximately 5–6 s). The tip of the finger has to be heated enough to maintain the evaporation of condensed water between the tip of the finger and the micro-object on the upper side of the released micro-object.
- Gripping: Two meniscuses have to be created: one between the glass plane and the bottom micro-object, and one between the upper micro-object and the tip of the gripper. The contact area between both objects has to be free of water when gripping. This is achieved by heating both Peltier elements above the dew point. This is done to ensure that the condensed water on the finger’s tip, on both micro-objects, and on the glass plane evaporates. Evaporation occurs in 3–4 s. Therefore, the capillary force is eliminated between all the parts. Then the temperature is decreased below the dew point on both Peltier elements. First, water is condensed on the tip and on the glass plane. Then, a water meniscus forms between the finger’s tip and the upper micro-object. At the same time, a water meniscus forms between the glass plane and the lower micro-object. This occurs when enough water is condensed on both the finger’s tip and the glass plane (the glass plane or objects or golden rod are seen blurred through the microscope because of the dew). This takes 2–3 s. After this time, a water meniscus between both micro-objects cannot form for 5–6 s. This is the time-window when only the upper micro-object is gripped, and can be reliably detached from the lower micro-object.

**Figure 6:**
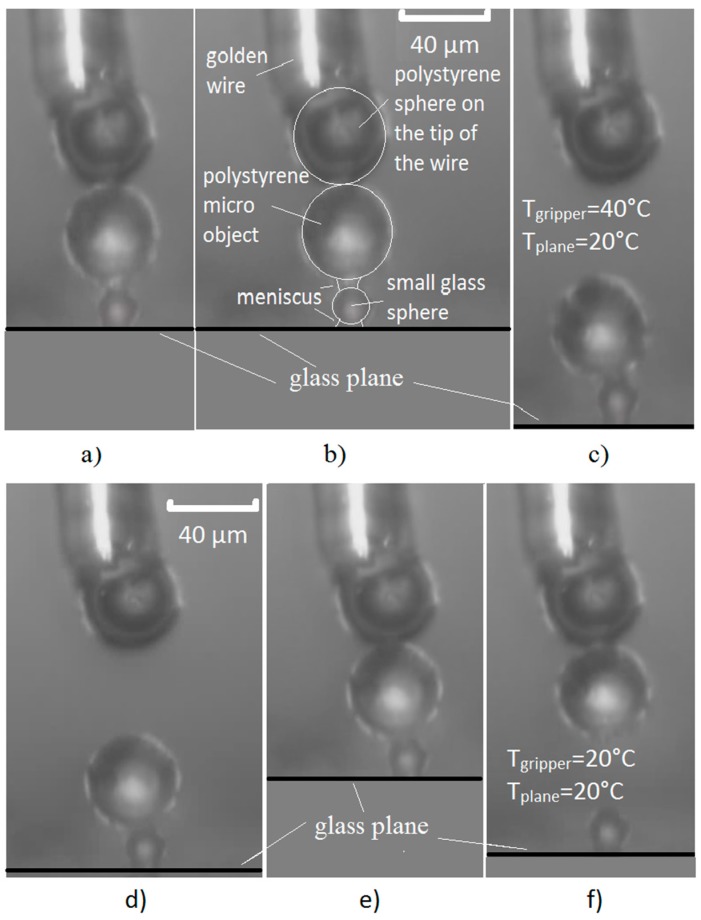
This figure shows the gripper gripping a polystyrene sphere with a diameter of 40 µm from a glass sphere with a diameter of 10 µm and releasing it from the gripper’s tip onto the top of a smaller glass sphere. A description of the photos is as follows: (**a**) the griper’s tip, with an attached polystyrene micro-object, is moved into contact with a smaller glass sphere; (**b**) a water meniscus is created between the plane and the smaller sphere, and between the smaller sphere and the polystyrene micro-object (larger sphere); (**c**) the gripper’s tip is moved away from the polystyrene micro-object; (**d**) a thin film of condensed water is created on the tip of the gripper and on the glass plane, forming water meniscuses between the glass plane and the smaller sphere, and between the gripper’s tip and the larger sphere—the contact between both spheres is still without a meniscus; (**e**) the polystyrene micro-object is attached to the tip of the gripper using capillary force; (**f**) the polystyrene micro-object (larger sphere) is detached from the smaller glass sphere.

Releasing and gripping a sphere on top of another sphere is presented in Figure 6. Figure 6a–c show a large sized micro-object (a polystyrene spherical object with a diameter of 40 µm) being released from the gripper and placed on top of a smaller micro-object (a glass spherical object with a diameter of 10 µm). The smaller object is on a glass plane. Figure 6d–f show the gripping of a large micro-object, which is on the top of a smaller micro-object, and its removal from the smaller micro-object. The smaller micro-object had not been moved from its place on the glass plane. The dew point in this particular experiment was approximately 23 °C at a pressure of 1 bar with a relative air humidity (RH) = 58%. The humidity was measured immediately before the experiment was executed. The temperature set-points for the Peltier elements were: 20 °C for the temperature below the dew point, and 40 °C for the temperature above the dew point. The time needed to cool-down the temperature from 40 °C to 20 °C, or vice versa, and condense/evaporate the thin layer of water, was 2–3 s. The temperature of the air was 28 °C. The complete releasing/gripping experiment described above is shown in Video S3.

### 3.2. A 3D Construction Made up of Micro-Sized Spheres

**Figure 7:**
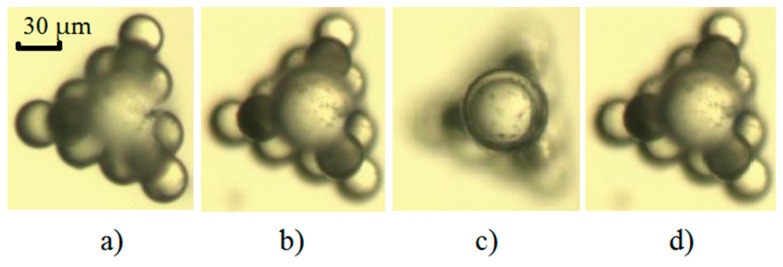
3D micro-object—a trident base with a glass sphere on the top: (**a**) the bottom layer; (**b**) the second layer; (**c**) the top layer; (**d**) the complete three-layered 3D construction.

Figure 7 shows a 3D object built using the above-described method. This is a three layer construction used as a trident base for the top glass sphere. The first layer—a bottom layer triangle made by ten light coloured polystyrene spheres (with diameters of 30 µm) is shown in Figure 7a. The second layer, made up of three darker polystyrene spheres (a triangle with a hole in the middle) is shown in Figure 7b. The polystyrene spheres of the first two layers are fastened together using sintering. Sintering the polystyrene spheres was done in hot air at a temperature of 170 °C for six and a half minutes, to produce a rigid connection. The third layer, made up of a single glass sphere with a diameter 50 µm, is shown in Figure 7c. The complete three-layered system (top view) is shown in Figure 7d. The photos in Figure 7 were made using an optical microscope. A similar 3D construction, showing the releasing of the spherical micro-objects in three layers in the vertical dimension, is shown in Video S4.

## 4. Discussion

### 4.1. The Influence of the Dew Point on the Measurement of the Pull-Off Force

Figure 8 shows the influence of the object’s dimension (sphere diameter), material and dew point on the pull-off force. The measurements of pull-off forces started with temperatures above the dew point (Figure 8, pull-off force at 30 °C). The temperature of the glass plane (table) on the *z*-axis was slowly decreased until it reached the dew point (the pull-off force, or van der Waals force was more or less constant). Then the pull-off force was increased (Figure 8 right). The lower the table temperature became, the more moisture accumulated between the objects (larger meniscus between objects), and a higher pull-off force was produced. This was the effect of the capillary force between the objects. We can see that the dew point was approximately 15–25 °C, depending on the air humidity at a pressure of 1 bar. The values of air temperature (*T_air_*) and RH (see Figure 8) were measured at the beginning of the experiment. The RH values in the chamber varied by about 10–20%, depending on the cooling and heating of the table during the experiment. The ratio of pull-off forces between the van der Waals force (temperatures higher than the dew point with no water layer between the objects) and the capillary force (lower than the dew point, where a thin layer of water exists between the objects) was about 2–6. This high ratio made gripping and releasing objects more reliable. The values for the temperature of the dew point *T_dpt_* were calculated at the beginning of the experiment from the measured values of RH and *T_air_*, using David Bolton’s equation [22]. The dew point temperatures in the micro-location for the gripping/releasing procedure could not be calculated because we could not measure the dynamic transient RH, nor the temperature values in the micro-location. The real dew point temperatures in the micro-location can be seen in Figure 8, where the pull-off forces start to rise from the minimal values.

The pull-off forces shown in Figure 8 were measured ten times for a single working point, then the average values and their standard deviations were calculated.

### 4.2. Comparison of the Measured Pull-Off Force with the Calculated van der Waals and Capillary Forces

The measured pull-off forces were checked by Equations (2)–(6). Equation (2) determines the capillary force between two spheres with Rabinovich’s model and Lambert’s correction [23]:

(2) $$F_{cap-sp/sp}=-\frac{2\pi R\gamma cos\theta }{1+\left(a/2d_{sp/sp}\right)}$$

where

(3) $$R=\frac{2R_{1}R_{2}}{R_{1}+R_{2}},\;d_{sp/sp}=\frac{a}{2}\left[-1+\sqrt{1+\frac{2V}{\pi Ra^{2}}}\right],\;2\cos\theta =\cos\theta_{1}+\cos\theta_{2}.$$

The capillary force between a sphere and a plane is given by [24]:

(4) $$F_{cap-sp/pl}=-\frac{4\pi R_{1}\gamma cos\theta }{1+\left(a/d_{sp/pl}\right)}$$

where

(5) $$d_{sp/pl}=a\left[-1+\sqrt{1+\frac{V}{\pi R_{1}a^{2}}}\right]$$

*R*_1_ and *R*_2_ are the radii of the spheres, *a* is the distance between the spheres, *γ* = 74 mN/m is the surface tension of the water, *V* is an estimated volume of the water bridge (meniscus) between the spheres, and *θ*_1_ and *θ*_2_ are the contact angles [25].

Equation (6) calculates the van der Waals forces between two spheres and between a sphere and a plane [8]:

(6) $$F_{vdW-sp/sp}=\frac{-AR_{1}R_{2}}{\left(R_{1}+R_{2}\right)6l^{2}},\;F_{vdW-sp/pl}=\frac{-A\cdot R_{1}}{6l^{2}},$$

where *A* is the Hamaker coefficient, and *l* is the distance of separation in the presence of nano roughness on the micro-sized objects [12,26] between the spheres, or between a sphere and a plane.

The calculated values for the capillary force *F_cap_* and the van der Waals force *F_vdW_* were compared with the margin measured values of the pull-off forces (the values on the right side of Figure 8 were compared with *F_cap_*, and the values on the left side of Figure 8 were compared with *F_vdW_*) (see Table 2 and Table 3). All necessary coefficients and variables used for the calculation of the capillary and Van der Waals forces are also shown in Table 2 and Table 3. The cause of the greater deviations between *F_vdW_* and *F*(30 °C) lies in the fact that the distances *l* (Van der Waals radius plus nano-roughness of the material’s surfaces) were not correctly measured. For example, different sources for distance *l* between the same materials (SiO_2_) differ from 0.15 nm to 0.4 nm. Our measurements performed in [26,27] gave us the value for the distance *l*.

### 4.3. Reliability of the Releasing Procedure

Releasing experiments were successfully carried out by placing a SiO_2_ sphere (with a diameter of 30 µm) onto a SiO_2_ plane. These experiments were repeated 50 times. The temperature of the tip was set to 30 °C, the temperature of the plane was set to 4 °C, the temperature of the air was 22 °C, the height of the water layer on the plane was estimated to be 1 µm, and the pressure of the air was set to 1 bar during the releasing procedure. 98% (49) of the releasing experiments were done without problems, while one experiment (2%) needed to be repeated once.

The experiments showing the reliability of releasing a SiO_2_ sphere (diameter is 30 µm) on the top of another SiO_2_ sphere (diameter 25 µm) were repeated 50 times. The conditions were the same as the procedure stated in the previous paragraph. 94% (47) of the releasing procedures were done without a problem, while the remaining three (6%) needed to be repeated from one to three times.

The reason for the unsuccessful releasing procedures was due to vibrations from the environment (mechanical shock or oscillations). The main source of oscillations was the vibrations from the water pump needed for cooling the Peltier elements. The oscillations were always present, and their frequency varied from 90 to 100 Hz with amplitudes of 0.1 to 0.5 µm. These measurements were performed on the top of the horizontal Peltier element. It appeared that the mechanical vibrations sometimes had resonant frequency values that were exactly the same as the spheres, or the constructions made out of the spheres. When this happened, the sphere which was still in contact with the finger’s tip and the releasing plane (or other objects) rotated for a few degrees (see Video S4—releasing a sphere in the third layer). This rotation prevented the accurate and reliable releasing of objects. When this effect occurred, the water pump was switched off to eliminate the oscillation. After this, the temperature of the Peltier element remained unchanged for a minute.

### 4.4. Accuracy of the Releasing Procedure

The reference position is the contact point between the released object and the plane during the releasing procedure. The reference position was measured before the capillary water-bridge (meniscus) between the object and the tip of the one-finger gripper disappeared (evaporated). The tip of the one-finger gripper was then moved away from the object (see the releasing procedure in Section 2.3). After that, the glass plane was heated up, and the water meniscus between the object and the plane evaporated. The actual position of the released object was measured after the evaporation of both meniscuses. The releasing accuracy was the difference between the reference and the actual position of a released spherical object with a diameter of 30 µm (polystyrene sphere) on the glass plane. The procedure was repeated 30 times to verify its accuracy. In all 30 tests, there were no differences detected between the reference and the actual position. Therefore, we concluded that the releasing accuracy was lower than 0.5 µm, because the resolution of the optical microscope was also 0.5 µm.

### 4.5. Influence of the Water Layer Thickness

The reason for the accuracy of releasing procedure lies in the fact that the releasing of the micro-sized objects was done onto a thin layer of water. The best results (reliability and accuracy during the releasing procedure) were achieved when the height of the water layer was at least 10 times smaller than diameter of the spherical object.

This newly developed method was successfully tested on objects that were between 5–60 µm. If the objects were larger, up 300 µm in diameter, then the time to accumulate enough water from the air took longer (15 s with low humidity). On the other hand, if the micro-sized object was smaller (with a diameter of 5 µm), then the accumulated moisture from the air produced a layer of water so high that the object was submerged in less than 5–6 s. This problem was solved by decreasing the air pressure to 1 mbar. This reduced the height of the water-layer, making it much smaller than the micro-object.

The thickness of the water layer on the objects during the gripping/releasing procedure, had an effect on the capillary force between the objects. If there was no water, then the capillary force did not exist. Only the Van der Waals force with a layer of air between the objects remained. This stage is seen in Figure 8, on the left side of the graphs, when the pull off-force is low. When the objects are blurred, due to condensation, then the pull-off force starts to increase. It increases until the meniscus volume becomes too high or the meniscus freezes. Approximately 2 s (at RH = 40%) are needed after the temperature of the object has decreased below the dew point temperature, and enough water has condensed (the objects are blurred). If the RH is greater than 40%, then the time of 2 s is decreased, and vice versa. If the temperature is below the dew point and does not reach the freezing point, or if it lasts, by mistake, for 1 min, then the object becomes completely submerged in the condensed water, thus stopping the capillary force. Now, only the Van der Waals force is left between the objects, in a medium of water. This force is remarkably lower than the Van der Waals force when there is a medium of air (in a vacuum) between the objects. This is due to the different Hamaker coefficients (A). The ratio of the van der Waals forces for polystyrene (PS) is 7.5. This is because A across the water for PS = 13 zJ, and A across a vacuum for PS = 98 zJ (see Equation (6)). Similarly the calculated ratio for SiO_2_ (quartz glass) is 41.2, because A across the water for SiO_2_ = 1.6 zJ, and A across a vacuum for SiO_2_ = 66 zJ.

The gripping/releasing method was successfully performed a few hundred times, mostly with micro-sized objects (5–60 µm). The gripping/releasing experiment of a glass micro-sized sphere with a diameter of 5 µm is shown in Video S5. The materials of the objects were SiO_2_ or polystyrene. The shapes of the objects were mostly spherical, but the method also works for irregular shapes (dust particles—see Video S6). The method was tested at an air (medium) pressure of 1 mbar to 1 bar, at a relative humidity (RH) between 30% and 60%, at an air temperature from 19 °C to 28 °C and at a dew-point temperature between 5 °C and 15 °C. The temperature of the Peltier elements (glass plane and golden tip) was changed from −3 °C to 40 °C. The thickness of the condensed water layer was estimated to be between 0.5 µm to 3 µm. The main drawback of the presented method was that if the RH values and temperature of the air were changed, then a new calibration of the dew point temperature was needed. But this was required once a day on average.

## Figures and Tables

**Table 1:** Overview of micro-assembly approaches.

Object Manipulation Characteristics					
Size of Object	Gripper Type	Approach	Dominant Gripping Force	Dominant Releasing Force	Ref.
Sub millimetre	2 + F	Not reviewed	-	-	-
	1F	Vacuum tool	Pneumatic/sucking	Gravity or pneumatic/blowing	[3,4]
		Variable curvature micro-gripper	Capillary/liquid drop	Gravity with shape based reduction of capillary force	[2,5]
		Capillary force gripping	Capillary/liquid drop	Gravity with cut-off of liquid meniscus	[1,6]
		Method for manipulating micro component	Capillary/electro wetting	Gravity with electro wetting reduction of capillary force	[7]
		Capillary hydrophobic/hydrophilic gripper	Capillary hydrophobic/hydrophilic gripper	Gravity with shape based reduction of capillary force	[8]
	T	Freeze tweezers	Ice, mechanical coupling	Gravity	[9]
Micro	2 + F	CSFH silicon MEMS	Friction or mechanical coupling	Not known	[10]
		Nanostructured and non-adhesive surface	Friction or mechanical coupling	Van der Waals	[11,12]
	1F	Variable Van der Waals force	Van der Waals	Van der Waals	[13]
		Vacuum tool	Pneumatic/sucking	Van der Waals	[3]
		Single-probe capillary	Capillary/dropwise condensation	Inertial/vibrations	[14,15]
		Fluid droplet based	Capillary/electro wetting	Capillary/electro wetting	[16]
		Vacuum micro-gripping tool	Pneumatic	Inertial/vibrations	[17]
	T	Variable contact surface (cryogenic)	Ice, mechanical coupling	Van der Waals	[18]
		Optical tweezers	laser beam	-	[19]

**Table 2:** Comparison of the measured pull-off forces (*F*) with the calculated capillary forces (*F_cap_*).

Objects	Material	*θ*_1_, *θ*_2_ [°]	*a* [µm]	*R*_1_ [µm]	*R*_2_ [µm]	*V* [pl]	*F_cap_* [µN]	*F*(−3°C) [µN]	*Dev.* [%]
sp.-sp.	SiO_2_-SiO_2_	30, 30	1	25	30	0.235	6.6	5.8	−12
sp.-sp.	SiO_2_-SiO_2_	30, 30	1	25	20	0.235	5.7	5.6	−2
sp.-sp.	SiO_2_-SiO_2_	30, 30	1	25	27.5	0.235	6.4	5.1	−20
sp.-sp.	PS-PS	20, 20	1	15	15	0.200	4.4	4.0	−9
sp.-pl.	PS-SiO_2_	20, 30	0.5	15	-	0.200	9.6	10.7	+11
sp.-sp.	SiO_2_-SiO_2_	30, 30	1	15	10	0.235	3.5	3.1	−11
sp.-sp.	SiO_2_-SiO_2_	30, 30	1	15	12.5	0.235	3.9	3.8	−2
sp.-pl.	SiO_2_-SiO_2_	30, 30	0.5	15	-	0.200	9.2	7.0	−23

**Table 3:** Comparison of the measured pull-off forces (*F*) with calculated van der Waals forces (*F_vdW_*).

Objects	Material	*A* [zJ]	*l* [nm]	*R*_1_ [µm]	*R*_2_ [µm]	*F_vdW_* [µN]	*F*(30 °C) [µN]	*Dev.* [%]
sp.-sp.	SiO_2_-SiO_2_	66	0.375	25	30	1.2	0.7	−41
sp.-sp.	SiO_2_-SiO_2_	66	0.375	25	20	0.9	0.8	−12
sp.-sp.	SiO_2_-SiO_2_	66	0.375	25	27.5	1.1	1.1	0
sp.-sp.	PS-PS	98	0.242	15	15	2.2	2.3	+4
sp.-pl.	PS-SiO_2_	80	0.300	15	-	2.3	1.3	−43
sp.-sp.	SiO_2_-SiO_2_	66	0.375	15	10	0.5	0.9	+80
sp.-sp.	SiO_2_-SiO_2_	66	0.375	15	12.5	0.6	1.0	+66
sp.-pl.	SiO_2_-SiO_2_	66	0.375	15	-	1.1	1.1	0

## Supplementary Materials

The following are available online at www.mdpi.com/2072-666X/8/6/182/s1: Video S1: Gripping/releasing a sphere from/to a surface plane with the capillary force; Video S2: Measurement of the pull-off force; Video S3: Gripping/releasing a bigger sphere from/to a smaller sphere with the capillary force; Video S4: Three-layer construction with a capillary gripper; Video S5: Griping/releasing a spherical glass micro-sized object with a diameter of 5 µm; Video S6: Gripping/releasing an irregular shaped dust particle from/to a surface plane with the capillary force.
